# Prospect of Polysaccharide-Based Materials as Advanced Food Packaging

**DOI:** 10.3390/molecules25010135

**Published:** 2019-12-29

**Authors:** Aleksandra Nešić, Gustavo Cabrera-Barjas, Suzana Dimitrijević-Branković, Sladjana Davidović, Neda Radovanović, Cédric Delattre

**Affiliations:** 1 Vinca Institute for Nuclear Sciences, University of Belgrade, Mike Petrovica-Alasa 12-14, 11000 Belgrade, Serbia; anesic@vin.bg.ac.rs; 2 Unidad de Desarrollo Tecnológico, Universidad de Concepcion, Avda. Cordillera No. 2634, Parque Industrial Coronel, Coronel 4190000, Chile; g.cabrera@udt.cl; 3 Faculty of Technology and Metallurgy, University of Belgrade, Karnegijeva 4, 11000 Belgrade, Serbia; sdavidovic@tmf.bg.ac.rs; 4 Inovation Centre of Faculty of Technology and Metallurgy, University of Belgrade, Karnegijeva 4, 11000 Belgrade, Serbia; neda.radovanovic.85@gmail.com; 5 CNRS, SIGMA Clermont, Institut Pascal, Université Clermont Auvergne, F-63000 Clermont-Ferrand, France; cedric.delattre@uca.fr; 6 Institute Universitaire de France (IUF), 1 rue Descartes, 75005 Paris, France

**Keywords:** polysaccharides, food packaging, chitosan, alginate

## Abstract

The use of polysaccharide-based materials presents an eco-friendly technological solution, by reducing dependence on fossil resources while reducing a product’s carbon footprint, when compared to conventional plastic packaging materials. This review discusses the potential of polysaccharides as a raw material to produce multifunctional materials for food packaging applications. The covered areas include the recent innovations and properties of the polysaccharide-based materials. Emphasis is given to hemicelluloses, marine polysaccharides, and bacterial exopolysaccharides and their potential application in the latest trends of food packaging materials, including edible coatings, intelligent films, and thermo-insulated aerogel packaging.

## 1. Introduction

The food industry is one of the most important industry sectors with very diverse processes and products. In addition to the quality of raw materials and processing equipment, the inevitable part of the food industry is food packaging. Food packaging is a major business sector in the packaging industry and, as with a food alone, food packaging must meet high standards of quality and safety, as well as the requirements of governmental regulations and policies, to be successful in the market [1].

The selection of food packaging encompasses a variety of aspects, including the food itself, packaging materials, storage, transportation, and distribution [2]. Besides the basic function of the packaging, to ensure integrity, preservation, and safety of food product, many other aspects determine the functionality of the packaging [3]. Some very important aspects which offer numerous opportunities for innovation and new solutions in food packaging are user-friendly and eco-friendly trends. User-friendly packaging satisfies consumer desire for convenience, maximum food quality, and minimum packaging waste. Easy handling, easy opening and dispensing, as well as food preparation in the package are examples of packaging that provide convenience to the consumer. Providing the high level of food security and protection from interaction with surrounding atmosphere is one of the main properties that packaging must have. Prevention of gas (oxygen, nitrogen, and carbon dioxide), moisture, as well as aroma permeability, slow down and/or reduce deterioration and changes in the properties of packaged foods [4].

Materials that have traditionally been used in food packaging include glass, metals (aluminum, foils, and laminates, tinplate, and tin-free steel), paper and paperboards, and plastics. These materials are significantly related to environmental pollution impact and to manufacturing requirements for nonrenewable resources.

The growing food industry that still applies traditional food packaging directly contributes to the increase of packaging waste. However, there is an increasing awareness among consumers worldwide that conventional packaging, especially plastic products, although very useful, create huge damage to the environment, water resources, and the entire ecosystem. Consequently, besides other strategy for reducing food packaging waste, such as source reducing, recycling, and reusing, the alternative packaging materials that are eco-friendlier are now required more than ever [5].

Biodegradable and/or bio-based polymers as new packaging materials are the most extensively explored in this context. Biodegradable refers to the ability of materials to break down and return to nature within a short time after disposal [6]. Biodegradable materials are made from both natural and fossil resources and are biodegraded by microorganisms in their natural environment. The polylactic acid (PLA), and the polyhydroxy butyrate acid (PHB) are two of the biopolymers targeted for use in the food industry as biodegradable packaging material. Bio-based polymers are made from bio-based resources, which may be extracted directly from plants or marine organisms such as polysaccharides and proteins, or produced by microorganisms in fermentative processes, such as microbial polyesters (PHAs) or microbial polysaccharides.

**Figure 1:**
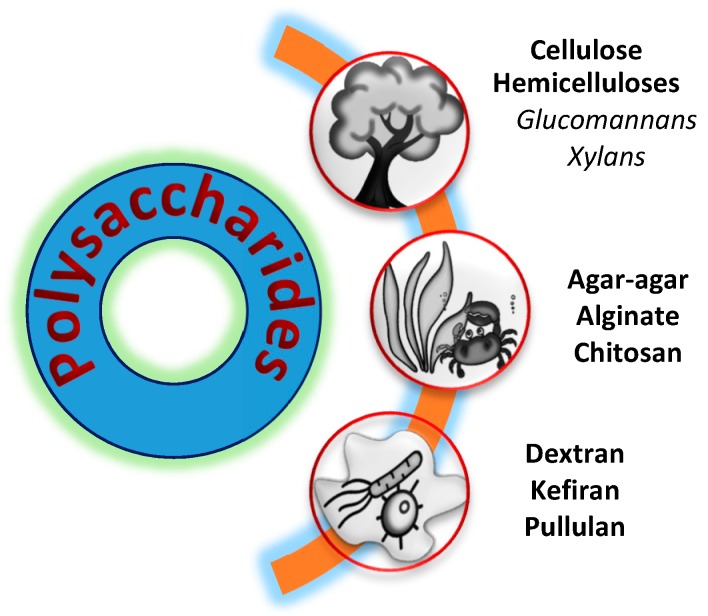
Examples of bio-based polysaccharides (from plant, marine organisms and microbial) as materials for food packaging.

Polysaccharides are the most abundant raw materials since they originate from plant or marine organisms’ biomass [7]. Microbial fermentation, on the other hand, represent engineered bioprocess that can provide metabolic product with desired properties and functionality [8]. Some of the most tested and applied polysaccharides for use in food packaging are shown in Figure 1.

Polysaccharides are the group of polymeric carbohydrates with inherent characteristics such as biocompatibility, biodegradability and non-toxicity towards living organisms [9]. These properties give them an advantage for use in food packaging, mainly in the form of edible coatings and films. Recently, polysaccharides have been also investigated to obtain bioactive and sensor materials as a component of active and intelligent packaging. Their use as primary packaging can potentially replace conventional packaging materials, partially or totally, which can reduce the overall use of synthetic materials [10,11]. Due to their protective functions, inherent or designed with the addition of antimicrobial, antioxidant, or other biologically active components, polysaccharide-based materials may simplify the total packaging structure [12,13].

Biopolymers have multiple film-forming mechanisms. Some mechanisms include electrostatic, hydrophobic or ionic interactions or other intermolecular forces such as covalent bonds (e.g., disulfide bonds and crosslinking). To be suitable for food packaging applications, the preparations of these films should include conditions and processes appropriate to the food process—namely pH modification, salt addition, heating, enzymatic modification, drying, use of food-grade solvents, or reactions with other food-grade chemicals [14].

However, some characteristics of polysaccharide-based coatings and films such as poor water vapor barrier and low mechanical properties slows down or prevents their mass/industrial applications [15]. Fortunately, depending on the final purpose of their use, natural properties of polysaccharides can be enhanced by structural modifications. Modifications and functionalization of polysaccharides are often necessary to achieve molecular structures of interest. Some possibilities for polysaccharide modification, to obtain more favorable characteristics for use in food packaging, include combination of two or more biopolymers or combination with some other component [16,17,18]. It is well known that a combination of more than two packaging materials is sometimes necessary to provide the best packaging solution for certain food products.

The introduction of a new reactive group will not change fundamentally the backbone of the macromolecules but will unlock advanced modifications required in specific applications [9]. Currently, it is common to apply polysaccharides in the preparation of bio-nanocomposites. Bio-nanocomposites are biopolymers which have been stabilized using nanoparticles. The nanoparticles enhance technical properties, such as barrier, thermal, chemical or mechanical stability and may include nanoclays and nanosilver [10,19].

The possibility of using some polysaccharides of plant, marine and microbial origin as well as their various form of application for advanced food packaging are shown in this manuscript, with the emphasis on the latest innovations in food bio-packaging technologies and on the review of published papers in last 5–8 years.

## 2. Latest Food Packaging Innovations

Most of polysaccharides are commonly used in the food industry and are approved by the FDA, which make them completely suitable, non-toxic, and safe basic components to process bio-based materials for use in the food packaging sector. Due to great film-forming and gel-forming properties, a variety of different polysaccharides have been used to produce thin membranes-films and gels for application in food, medicine, and pharmacy. Beside this, the latest innovations in food packaging technologies include development of polysaccharide-based edible films, intelligent packaging films and aerogels in order to maintain the quality and safety of food products during storage and transportation and to provide the extension of shelf life. Moreover, additional role can be beneficial in terms of food protection from environmental influences such as heat, light, presence or absence of moisture, oxygen, enzymes, dust particles, gas emission, etc. Generally, polysaccharide-based materials present good barrier properties toward oxygen and carbon dioxide at low and moderate relative humidity. However, their main drawback is poor barrier against water vapor due to hydrophilic nature and poor mechanical stability, which can be overcome by blending with another polysaccharide or lipids and by introduction of biologically active components in the system. The following sections will describe new bio-based food packaging technologies and the review of different classes of polysaccharides with potential application in targeting the food packaging sector.

### 2.1. Edible Food Packaging

**Figure 2:**
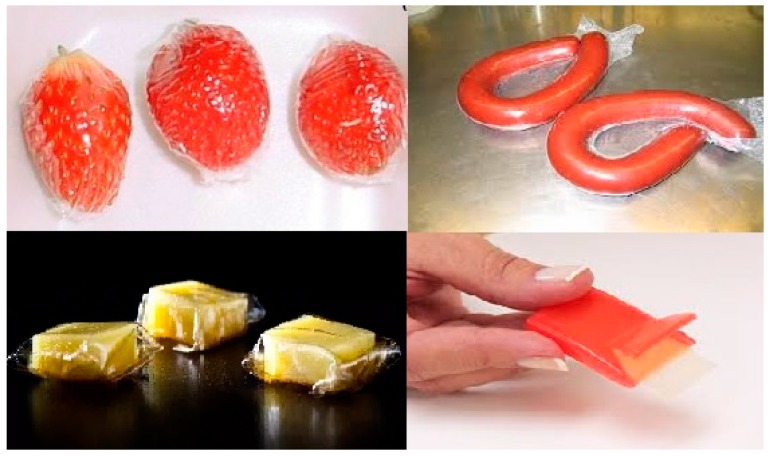
Some examples of edible films in the food packaging industry.

Edible food packaging includes thin films and coatings that can be directly applied on food products or being used as a food wraps, providing several important functions, such as the prevention of oxygen migration, water, carbon dioxide, lipids, and flavoring ingredients in the food product. Hence, an edible film has protective barrier role to extend the shelf life of food products. However, it is important that the packaging must not be absolutely water, gas, and ethylene barrier proof, because fruit and vegetables have a certain degree of respiration. Moreover, the films and coatings must adhere well with the food product and should not have any mechanical damage (Figure 2).

Materials used to produce edible packaging are mainly soluble in water. The use of organic solvents is not recommended, due to possible contamination of food products and increased toxicology issues. It is important to highlight that such material should not have a negative impact on human health and must be approved by official food safety institutions. In addition, by being edible and from renewable sources, this type of packaging represents a unique category of materials that differ from other conventional packaging materials. Since polysaccharides are food-grade chemicals, commonly used in the food sector and soluble in water, they become suitable candidates as a raw material to process edible packaging films and coatings.

Polysaccharide-based films and coatings are colorless, have an oil-free appearance and a minor caloric content and can be potentially applied to prolong the shelf life of fruit, vegetables, shellfish, or meat products by significantly reducing dehydration, darkening of the surface, and oxidative rancidity. In addition, certain polysaccharides in the function of edible packaging can serve as carriers of some organic acids that inhibit the growth of bacteria as potential food contaminants (*Listeria monocytogenes*, *Salmonella*, and *Escherichia coli*). Besides, the polysaccharide edible films can act as antioxidants, but also as a functional supplement (nutraceuticals) to the product itself. Apart from being environmentally friendly, many of these new edible materials can also be a good source of vitamins or minerals, but also can improve the taste of food products [20,21].

### 2.2. Intelligent Packaging

**Figure 3:**
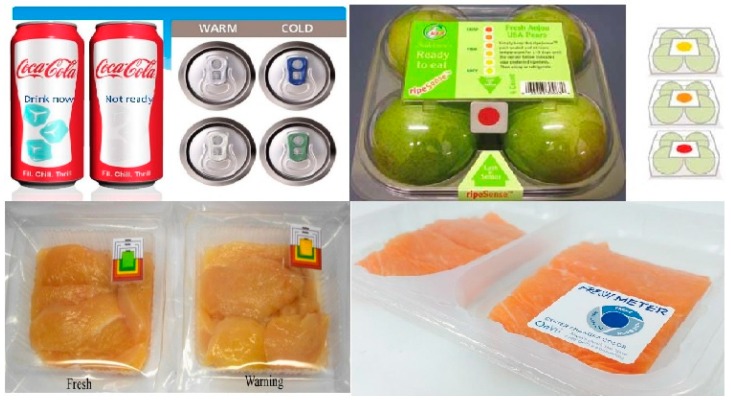
Some examples of intelligent bio-based food packaging.

Intelligent packaging systems are materials that come into contact with food, monitoring and providing the condition of packaged foods in terms of the quality and freshness of products during transport and storage. Generally, intelligent packaging contains an external or internal indicator which is fixed to the surface of packaging or headspace and gives specific qualitative information about the food. The most investigated types of indicators for intelligent packaging are gas indicators, time–temperature-sensitive indicators and freshness indicators (see Figure 3).

Gas indicators are specifically important in packaging of the food that is capable of respiration, which significantly influences the atmosphere inside the package. The gas composition within a package can easily change due to the interaction of food with its environment. The principle of action of oxygen and carbon dioxide indicators is based on color change caused by chemical or enzymatic reactions. They usually come in the form of a label or tablet, and the greatest advantage of their application is that they show the quality of the packaged product. Specifically, it is not necessary to open the package to check the quality. There is also the possibility to control unwanted microorganisms by installing or dragging the quaternary ammonium salt.

The temperature indicators indicate (usually in the form of mechanical deformation, color change or color migration) the heat loads that the packaged product is exposed to in the distribution chain. In this case, there are two types of indicators: continuous indicators which records the temperature changes (weather-temperature indicators) and registration indicators of extreme conditions i.e., whether the product was exposed to higher or lower temperatures than the critical temperature. Commercial indicators of the time–temperature type operate based on chemical diffusion, polymerization reaction, or enzymatic reactions. This type of indicator is mostly used in packaging, transporting, and storing the frozen food.

Freshness indicators have a role to monitor the microbial breakdown of products in the headspace of packaged food products, mainly fish and meat. Generally, pH indicator dyes that are sensitive to volatile compounds associated with food product spoilage are used. This pH-sensitive dye is entrapped within a polymeric matrix, and when the spoilage volatile compounds are released in the package, visible color changes can be observed as a response [22,23,24].

In recent years, numerous polysaccharide-based materials have been investigated as bio-indicators for intelligent packaging, to promote eco-sustainability and green technology. In addition, various different bioactive and temperature/pH-sensitive components, such as natural dyes and extracts have been exploited to evaluate their efficiency and applicative potential to replace commercially used synthetic and toxic compounds that serves as indicators incorporated in polymer matrix. The principle of work of polysaccharide/bioactive component indicators are the same as for synthetic ones. Bioactive components are usually incorporated into polysaccharide matrix and final material is obtained by casting method or lyophilization. The bio-indicators have been investigated in different forms: as an insert pad inside commercial package, or as an edible film indicator that is coated directly on food product [25,26,27,28].

### 2.3. Aerogels in the Food Packaging Sector

Aerogels are a class of lightweight and highly porous solid materials that possess large surface area, high mechanical stiffness, and low thermal conductivity. Generally, aerogels are prepared by replacing the liquid from gel with air by CO_2_ supercritical drying. Specifically, the solvent is removed from the gel using compressed CO_2_ at operational conditions above the critical temperature and pressure of the solvent-CO_2_ mixture. In this way, vapor-liquid interfaces are avoided by homogenous single-phase mixing process. The presence of supercritical fluid in the pores of gel, without liquid phase, prevent the appearance of surface tension, thus inhibiting the pore collapse during drying. After complete substitution of the liquid solvent in gel with supercritical CO_2_, the CO_2_ can be easily released by slow depressurization at operational conditions above its critical temperature. Hence, this drying technique allows complete preservation of the gel’s three-dimensional structure without pore collapse [29,30,31].

**Figure 4:**
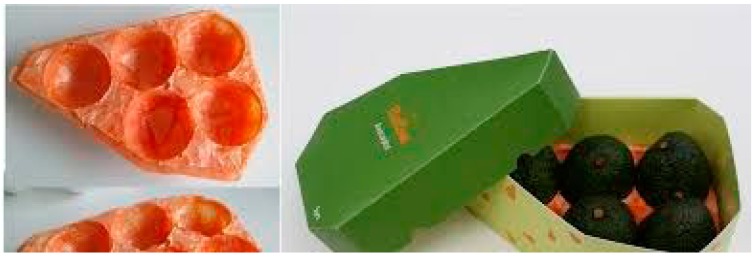
Potential aerogel food package [39].

Although aerogels have been widely exploited and investigated in food, medicine, and drug delivery application, due to high surface area, porosity and consequently easier loading of bioactive components/nutrients/drugs, the research focused on the development of polysaccharide-based aerogels has been recently started. Because of their biodegradability, biocompatibility, and edibility, polysaccharides have been more investigated in last decade to obtain multifunctional bio-aerogels that could potentially replace synthetic ones in targeted applications [32,33,34,35]. One of the latest innovations is use of aerogels as a part of active food packaging, where polysaccharide-based aerogels can be inserted into package and used as bio-absorbers of humidity or oxygen [32,36]. In addition, they can be carriers for nutrients and bioactive components to hinder or completely inhibit microbiological contamination of food products. Polysaccharide aerogels possess low thermal conductivity, in the range of 16–22 mW/mK [37], which is comparable to silica aerogels and synthetic polystyrene foams, currently used in packaging of hot or chilled food and beverage. Hence, polysaccharide-based aerogels can be used as mechanical supporters in bio-food packaging of thermal-sensitive food [38]. Recently, it has been investigated fully bio-based aerogel package for different fruit models with special design (see Figure 4).

## 3. Case Studies of Polysaccharides and Their Role in Food Packaging

### 3.1. Plant Polysaccharides

#### 3.1.1. Cellulose

**Figure 5:**
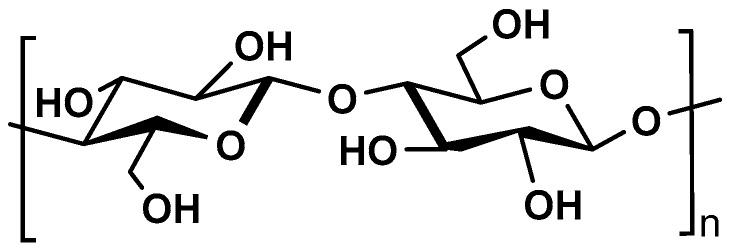
Chemical structure of cellulose.

Cellulose is the most abundant biopolymer in the world. It is found in cell walls of all plants, but also in some fungi and algae, in some marine organisms of tunicates family, invertebrates, and some Gram-negative bacteria [40]. Cellulose is a linear homopolysaccharide composed of *d*-glucopyranosyl units linked by *β*-(1→4) glycosidic bonds (Figure 5).

The most commercially exploited sources of cellulose are wood pulp and cotton fibers [41]. However, recent studies have shown that various plant-based waste materials such as peel, husk and shell, and sugar cane bagasse present suitable sources of cellulose, which is important from both economic and ecological point of view [42,43,44].

Cellulose has good film-forming performance, high chemical stability, and it is possible easily to synthetize cellulose derivatives [45]. However, application of cellulose to obtain edible films is limited due to its insolubility in water and vast majority of organic solvents [46]. This drawback could be overcome by derivatization, which significantly improves not only water solubility, but also thermoplastic behavior of cellulose.

Cellulose derivatives used for preparation of edible food packaging include ethers (methylcellulose, carboxymethyl cellulose, hydroxypropylmethyl cellulose, and hydroxypropyl cellulose) and esters (cellulose acetate) [41]. Otoni et al. showed that higher substation degree and molecular weight of hydroxypropyl methylcellulose improved significantly mechanical and water vapor barrier properties of cellulose-based edible films [47]. In addition, hydroxypropyl methyl cellulose and methyl cellulose edible coatings showed to be efficient in reduction of moisture loss and fat uptake of deep-fried starch products [48]. To extend the functionality of cellulose derivative-based edible films, various antioxidant and antimicrobial components were incorporated in the system. For example, the biological activity of carboxymethyl cellulose films was improved by addition of dipalmitoyl lecithin liposomes loaded with quercetin and rutin [49], *α*-tocopherol [50], spent coffee ground polysaccharides [51], candelilla wax [52]. Dicastillo et al. obtained high antioxidant activity of methyl cellulose/murta fruit extract and methyl cellulose/native Chilean berry extract films, which makes them suitable for food packaging of fatty food products [53,54]. Tumbarski et al. [55] investigated the effect of carboxymethyl cellulose edible coatings with a bacteriocin from Bacillus methylotrophicus BM47 on the shelf life of fresh strawberries. Strawberries were coated with 0.5% CMC and 0.5% CMC with bacteriocin and stored at 4 °C and 75% RH for 16 days. The results showed the impact of bacteriocin on fungal growth inhibition, thus delaying the decay of fruit for 4 days more, when uncoated and CMC-coated strawberries were compared. Coated samples also exhibited lower weight loss, lower TSS amount, and maintained antioxidative activity throughout the storage period. These effects however were not affected by bacteriocin addition. To obtain coatings with antibacterial and antioxidant properties, Raeisi et al. [56] developed carboxymethyl cellulose (CMC) coatings incorporated with different concentrations of *Zataria multiflora* essential oil (ZMEO) and grape seed extract (GSE). The effect of these coatings on the rainbow trout fillets’ quality during 20 days of storage at 4 °C was investigated. The results showed that coatings were effective in preventing fish spoilage, since total volatile basic nitrogen values for coated samples were maintained below 25 mg *N*/100 g, which is considered the highest acceptable level [57], while control uncoated sample reached value of 36 mg *N*/100 g within 15 days of storage. Moreover, the coatings were effective in lowering the total viable count, lactic acid bacteria count, and Pseudomonas spp. count of rainbow trout fillets during refrigerated storage and the effect was more prominent with increasing the concentrations of ZMEO and GSE in CMC coatings. However, high concentrations of additives had negative effects on the sensory properties. Thus, coating formulations CMC + 1% *v*/*v* ZMEO + 0.5% *v*/*v* GSE and CMC + 1% *v*/*v* ZMEO + 1% *v*/*v* GSE were considered the optimal for attaining required antibacterial and antioxidant effects to prolong the shelf life of fish fillets during refrigerated storage. Another approach to obtain antimicrobial CMC-based coatings has been made by Saba and Amini [58]. They assessed the effect of ZnO nanoparticles in combination with CMC on ready-to-use pomegranate during 12 days’ storage at 4 °C. Pomegranate arils were subjected to different treatments, i.e., dipping in water (control) and 0.1 or 0.2% (*w*/*v*) nano-ZnO suspension, followed by coating with 0.5% (*w*/*v*) CMC. The results showed that coatings were effective in reducing total yeast + mold and total aerobic mesophilic bacteria counts during 12 and 6 days of storage, respectively. Coatings also reduced weight loss of arils with the most significant effect observed with CMC + 0.2% ZnO at 9 days of storage. The results of total phenol content, total anthocyanin, vitamin C, and antioxidant capacity determination revealed that nano-ZnO + CMC coating prevented bioactive constituent decline during aril storage, which suggested its potential to extend its shelf life.

Hydroxyethyl cellulose and cellulose acetate films enriched with resveratrol inclusion complex showed high antimicrobial activity toward *Campylobacter* [50], while nisin incorporated intohydroxypropyl methyl cellulose films proved high antimicrobial activity toward *Listeria*, *Enterococcus*, *Staphylococcus*, and *Bacillus* spp. [59]. Silva et al. [60] developed antimicrobial bilayer films based on hydroxyethylcellulose and cellulose acetate with resveratrol and its inclusion complex with hydroxypropyl-γ-cyclodextrin as active agents. The results of antibacterial activity against *Campylobacter* and *Arcobacter* spp. revealed higher inhibitory potential of resveratrol than resveratrol inclusion complex contained films. The potential of these active bilayer cellulose films to inhibit the growth of foodborne pathogens makes them suitable for food packaging of poultry meat. Rhimi et al. [61] studied the effect of cypress seed extract (CSE) concentration on the properties of HPMC films and assessed their ability to prevent photo-oxidation of virgin olive oil during accelerated storage. The results showed that incorporation of 2% CSE led to decrease of peroxide formation and delay oxidation of olive oil. The peroxide value of olive oil samples covered with control HPMC films was 10 times higher than those covered with films incorporated with 2% CSE after 11 days of storage.

Beside cellulose derivatives, one of the most interesting products is cellophane that is produced from regenerated cellulose. Cellophane is thin and transparent material with good barrier properties toward grass, oil, water, and bacteria, which make this material suitable in the food packaging industry. Specifically, cellophane has been commercially used in the food packaging of fresh fruit, vegetables, sandwiches, cookies, and bakery food since 1930.

Regarding sensors/indicators for intelligent packaging, cellulose, and its derivatives have been widely investigated. Zhang and Lim developed inject-printed cellulose indicators to monitor concentration of CO_2_ gas, which existence in packaging influences a quality of many food products [62]. Ezati et al. proved that cellulose/chitosan film that contain alizarin could efficiently monitor the freshness of minced beef during storage time [63]. Mills at el. developed temperature-activated humidity sensor based on hydroxypropylmethyl cellulose and thiazine dyes [64]. This sensor has ability to change its color within seconds at relative humidity lower than 70%, which makes it suitable in food packaging of dry food. Liang et al. obtained high pH and NH_3_ sensitivity of *Artemisia sphaerocephala Krasch*. Gum/carboxymethyl cellulose films enriched with red cabbage anthocyanins, which is suitable property for pH and gas/sensing labels in food packaging [65].

Cellulose is one of the most investigated biopolymers to produce aerogels. Generally, the synthesis of cellulose-based aerogels are consisting of three steps: (a) dissolving the cellulose or cellulose derivatives in organic solvent, (b) sol–gel step by agglomeration of colloidal particles (in the presence of crosslinker or by changing the temperature and pH) or by a phase separation process (in the presence of coagulant) and (c) supercritical drying. Cellulose aerogels possess high mechanical strength and thermal conductivity comparable with silicon aerogels [66]. Depending on the route of crosslinking, as well as on operational drying conditions, it is possible to tailor morphological and textural properties of cellulose aerogels [66,67,68,69,70]. Super water adsorbent aerogels that can be potentially used for food packaging application were obtained by regenerated cellulose [71] and cellulose nanocrystals [72]. On the other side, Oliveria et al. used nanocellulose obtained from *Gelidium sesquipedale* seaweed to reinforce PVA food packaging aerogels [73].

#### 3.1.2. Hemicellulose

Hemicelluloses are polysaccharides that are obtained by extraction from plant materials or as byproducts of various wood and plant processing technologies. The presence of lignin and ester and ether-linked lignin-carbohydrate complexes, as well as hydrogen bonds between polysaccharides in the plant cell wall, can restrict the isolation of most hemicelluloses [74]. They are very heterogeneous group of polysaccharides, with considerable differences in composition and structure, depending on the biological source and localization, even within the same plant. They can be linear or branched, and compared to cellulose, have shorter chains and lower molecular mass and are non-crystalline [74]. Hemicelluloses consist of *β*-(1→4)-linked backbone of either glucose, mannose, or xylose linked in equatorial configuration. Based on differences in the primary structure, four main groups of hemicelluloses can be identified: xyloglycans (xylans), mannoglycans (mannans), *β*-glucans, and xyloglucans. The presence and the distribution of side chains in hemicellulose is non-random and it determines the structure of the polymer, affecting its physico–chemical, rheological, and functional properties as well. Solubility, interactions with other cell wall components, degradability by enzymes, solubility, and gelling properties are highly influenced by the degree of substitution and substitution patterns. For example, substitution of the xylan chain can be the main factor that influences water solubility. Some arabinoxylans from certain cereals can form gels only after oxidation, while oxidation of cereal derived β-glucan increases their water solubility [74,75,76].

Generally, hemicelluloses possess good gelling and film-forming capacity. Major drawback is their highly hydrophilic nature that limits their application in native form, due to their low moisture resistance. However, numerous free hydroxyl groups distributed along the backbone and side chains of hemicellulose, enable its’ chemical functionalization and formation of derivatives with desirable solubility, crystallinity, film-forming ability, and hydrophobicity [73,77]. Esterification with acid chlorides or anhydrides, etherification, including carboxymethylation and alkylation, acetylation, as well as ring-opening graft polymerization with hydrophobic polymers have been successfully used for efficient tailoring of hemicellulose molecules with the resulting products having improved properties compared to the native compound [28,78,79]. Other possibilities for the hemicellulose functionalization include ion-mediated oxidation, hydroxypropylsulfonation and hydroxypropylation [78]. Considering food packaging application, the most investigated hemicellulose are xylans, glucomannans and *β*-glucans, thus only these 3 biopolymers will be further described.

##### Xylans

**Figure 6:**
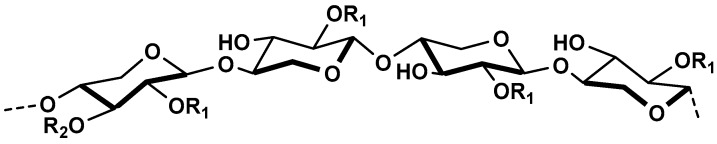
Main chemical structure of xylans. With R_1_ and R_2_ are H or different side groups.

Xylans are the most common hemicelluloses and they are considered to be the second most abundant biopolymer in the plant kingdom. Xylan polymer consists of *β*-(1→4)-d-xylopyranose backbone with different side groups attached with (1→2) and/or (1→3) linkages (Figure 6).

The type of branching from xylose backbone determines the sub-type of xylan. Arabinoxylans, typical for monocots and cereal grains, are primarily substituted with α-l-arabinofuranosyl unit on position C2 and/or C3 of xylose chain, but they can also be substituted with acetyl groups [75,76]. In dicots and hard woods, glucuronoxylans have a side chain on the C2 position of either *α*-d-glucuronic acid or its 4-*O*-methyl derivative. Like arabinoxylans, glucuronoxylans can be partially acetylated depending on the plant source and the degree of acetylation highly influences their solubility [75,77]. Xylans from softwoods, grasses and cereal straws are substituted with *α*-l-arabinofuranosyl unit and 4-*O*-methyl-α-d-glucuronic acid on O-2 and O-3 position of the xylose chain and are referred as arabinoglucuronoxylans. Arabinose residue can be linked via ester linkages by ferulic and cumaric acid. Molecular mass of xylans can vary greatly depending on the source (from 15 kDa in softwood to 300 kDa in some cereal derived xylans) and extraction method. The degree of polymerization ranges from 50–200 [78]. Woody materials, such as wood meal and shavings or forest chips and many agricultural crops and their residues, such as cereal straw, sugar cane, corn stalks and cobs, bran, hulls and husks from starch production represent good sources of xylan [75].

Mikkonen et al. developed xylan films with low oxygen and grease permeability, but low to moderate mechanical stability [78]. Moreover, in another paper they showed that by varying the degree of substitution of hydroxypropyl groups and concentration of plasticizer, could be obtained xylan films with good barrier and mechanical properties [79]. The low oxygen permeability of spruce xylan was also proved by Escalante et al. [80]. Ramos et al. used carboxymethylxylan and a 2-dodecenyl succinic anhydride-modified xylan to obtain paper/xylan laminates that can potentially replace packaging papers coated with polyethylene films [81]. They showed that paper/xylan laminates possess high mechanical and water vapor resistance, but moderate oxygen barrier.

Regarding the aerogels, there are only a few published papers related to physico–chemical properties of xylan-based aerogels, without specific food packaging application. Morphological and textural properties of arabinoxylan aerogels [82] and cellulose/lignin/xylan aerogels [83] have been studied.

##### Glucomannans

**Figure 7:**
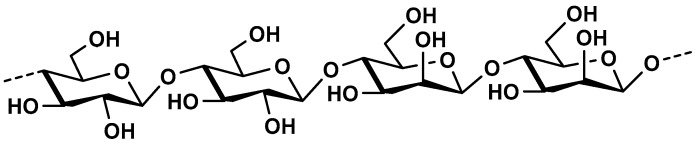
Main chemical structure of glucomannans.

Glucomannan belongs to mannan family of plant hemicelluloses, and it is present in the wood of conifers and in minor amounts in the wood of dicots (2–5%). The most common source of glucomannan is the tuber of konjac plant (*Amorphophallus* konjac) where it represents 60–80% of its dry weight. Glucomannan is also a constituent of bacterial and yeast cell wall. It can be potentially recovered as a by-product from various processes of the forest and agroindustry [78,84]. Glucomannan is mainly a linear-chain polymer that comprises of alternating d-mannopyranose and d-glucopyranose units connected with *β*-(1→4) linkages (Figure 7).

The occurrence of branching depends on the source [75]. In hardwood it is linear polymer, with no substitutions, and a degree of polymerization is approximately 60–70. In some case mannose unit can be substituted on C2 or C3 position, with α-d-galactopyranose via (1→6) linkages that are generally esterified with acetyl groups. These are usually present in softwood and are referred to as galactoglucomannans. The ratio of galactose:glucose:mannose can vary, and it is about 0.1:1:4, or 1:1:3 in galactose richer fraction. The degree of polymerization is ranging from 100–400. The ratio of *β*-d-mannose and *β*-d-glucose in konjac glucomannan is about 1.6:1 with side branches of *β*-(1→6) glucosyl unit attached to the C-3 positions of the chain. Small amounts of acetyl groups are also present in konjac glucomannans. Glucomannans are water-soluble, with molar mass varying from 30 kDa, reported for certain softwood derived glucomannans, to 1 × 10^6^ Da or higher, reported for konjac plant derived glucomannans [84,85]. High molecular weight of konjac glucomannans leads to very viscous solutions.

Kurt and Kahyaogly investigated physico–chemical properties of salep glucomannan edible films and compared their properties with locust bean gum and guar gum films [86]. Salep glucomannan films showed to have better oxygen and water vapor barrier but lower mechanical resistance than other investigated films. Due to high water solubility of glucomannan films and lower mechanical resistance, this biopolymer is usually combined with other polysaccharides such as curdlan [87], gellan gum [88], ethyl cellulose [89], or various nano/microfibers are incorporated into biopolymer matrix. Wang et al. showed that addition of polydopamine functionalized microcrystalline cellulose improved significantly mechanical and water vapor barrier properties of konjac glucomannan food packaging films [90]. Moreover, addition of chitosan/gallic acid nanoparticles and chitosan/nisinin to konjac glucomannan films gave one more advantage in terms of antimicrobial activity toward against foodborne pathogens *S. aureus*, *Listeria monocytogens*, *E. coli* and *Bacillus cereus* [91,92]. Zhang et al. investigated konjac glucomannan/carrageenan/nano-silica films for the preservation of the postharvest mushrooms [93]. Films containing 0.3% of nano-silica showed the best physico–chemical properties and tendency to extend the shelf life of fresh Agaricus bisporus from 5 to 12 days.

Regarding thermal-insulated biomaterials, glucomannan materials obtained by supercritical drying have not been reported in the literature, but cryogels obtained by freeze-drying have been widely investigated [94,95]. Incorporation of silica or starch/wheat straw into konjac glucomannan could positively influence the hydrophobicity, mechanical strength, and the thermal insulation property of final biomaterial, which are key parameters in insulated food packaging, i.e., packaging of temperature-sensitive food [96,97]. On the other hand, Lethonen et al. investigated galactoglucomannan/anionic cellulose nanofibrils aerogels that contained food-grade sunflower oil for in situ production and release of hexanal, to prolong the shelf life of blueberries and cherry tomatoes [98]. Hexanal was produced and released by use of enzymes or photoinitiators as catalysts. The released hexanal reached a value of 7–23 µmol/g of aerogel for a period of at least three weeks. Since one of the new bio-based active packaging concepts requires less than one gram of aerogel in one liter package to preserve fresh plant products against softening and mold growth, this work could improve the economy and sustainability of the food chain.

##### β-glucans

**Figure 8:**
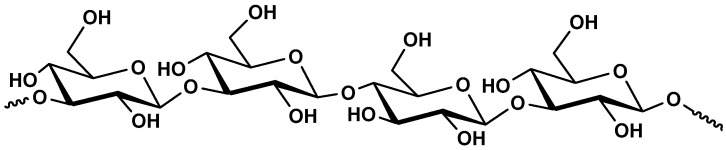
Main chemical structure of Cereal *β*-glucans.

*β*-glucans are polysaccharides that occur widely in nature and can be found in fungi, mushrooms, yeasts, algae, bacteria, and plants, mainly cereals [99,100]. This section will focus on *β*-glucans originated from cereals. In cereals, β-glucans are found in sub-aleurone and internal aleurone endosperm cell walls of the grains and their content can vary depending on the source. It is higher in barley (3–11%) and oats (3–7%) then in rye (1–2%) and wheat (<1%). Cereal *β*-glucans are linear homopolysaccharides composed of *β*-d-glucopyranosyl units linked with (1→3) and (1→4) glycosidic bonds (Figure 8).

They consist mainly of cellotriosyl and cellotetraosyl units and a minor amount of long *β*-(1→4)-linked cellulosic blocks with glucopyranosyl residues. The higher ratio of cellotriosyl/cellotetraosyl is associated with good water solubility and faster gelation tendency [101,102]. The source from which *β*-glucans are extracted and the nature of extraction procedure greatly influence their structure and molecular weight. Molecular weight can vary from 20 kDa to 40,000 kDa and it strongly influences physical and biological functions of *β*-glucans [99].

*β*-glucan coatings and films are generally physically stable with moderate mechanical and good gas barrier properties that can be used for protecting food from oxidation. The major drawback of using β-glucans in the food packaging industry is high cost of extraction and purification processes, as well as low water vapor barrier. Use of all natural polymer components, i.e., proteins and other cell wall derived polysaccharides such as arabinoxylans as blends with *β*-glucans, from the same endogenous source or other grains, can improve mechanical properties and water vapor barrier, but are more cost-effective [103,104,105]. Various modifications of *β*-glucan i.e., carboxymethylation, ion-mediated oxidation, and esterification have also been reported to have positive effect on the structural properties of *β*-glucan as well as physical properties of *β*-glucan-based composite materials. Comin and coworkers investigated widely *β*-glucan aerogels [106] that can serve as materials in food packaging or directly in the food sector for delivery of nutraceuticals, such as flax lignan [107], flax oil [108].

#### 3.1.3. Starch

**Figure 9:**
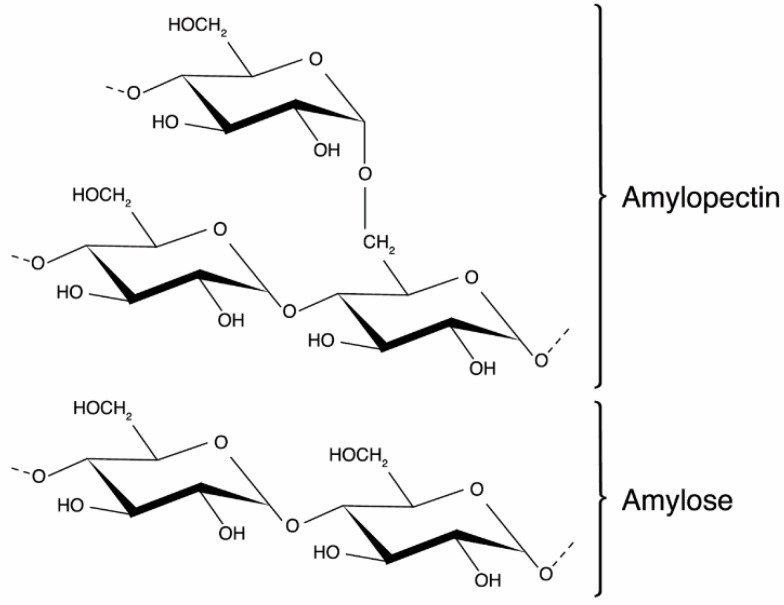
Chemical structure of starch polysaccharide.

Starch is a natural polysaccharide composed of amylose and amylopectin. Amylose is a linear polymer of α-1,4-linked d-glucose units. Amylopectin is a highly branched polysaccharide consisting of short chains of α-1,4-linked d-glucose units and 4–5% branch points with 1,6 linkages occurring periodically at every 20–30 anhydroglucose units [109]. Their chemical structure is presented in Figure 9. Most commercial starches are made from corn, although wheat, tapioca, rice, cassava, and potato starch are also used [110]. This polysaccharide is renewable, biodegradable, easy to modify, both physically and chemically, and is available at low cost [110]. These facts make it an appealing raw material for the manufacture of food packaging products.

A significant difference between starch and other polysaccharides is the property to convert to thermoplastic material when it is exposed to shear and high in the presence of plasticizers, i.e., water, glycerol and oils [111,112]. The obtained compound is known as thermoplastic starch (TPS), and it is one of the commercially existing starch products [112]. TPS could be processed by industrial methods (compression molding, extrusion, and blowing), to obtain starch derivatives or new products such as films, bags, food containers, clamshells, etc. [113].

On the other hand, starch films have been widely studied, since they are edible, odorless, non-toxic, tasteless, and colorless and act as proper oxygen barriers [110]. To be used as food packing material, starch films have two main disadvantages compared to synthetic polymers: moisture absorption susceptibility and poor mechanical performance [114]. Moreover, it does not have antimicrobial nor antioxidant properties. Different approaches to overcome these drawbacks have been studied. Some of them are related to novel biocomposite preparation, mixing starch with plant extracts, essential oils, and other natural products to provide biological properties [115,116,117,118,119,120,121]. Another method has been through the film reinforcement with organic or inorganic nanoparticles (Ag ZnO, CuO) [122,123,124], nanoclays [125] or carbon nanotubes [126]. If a complete biodegradable/compostable film or coating would be desired, starch can be blended with polysaccharides, proteins, biodegradable polymers (PVA, PVP), and biopolyesters (PLA, PHB, PHBV, PCL, PBS, PBAT) [112,118,122,127,128,129,130]. This approach allows the obtaining of novel biocomposites with improved properties that could be used as a carrier of different substances, releasing them in a controlled manner once in contact with foods [131]. Then the package is an active barrier that contributes to food preservation [121].

In addition, starch derivatization is another way to improve film properties because chemical modification reduces matrix polarity due to the –OH groups substitution with highly hydrophobic groups [113]. Polysaccharide hydrophobization can reduce the moisture absorption capacity and improve the compatibilization with hydrophobic matrices. An example of starch derivatization reactions can be succinylation [132], fatty acid modification [113] or polymer grafting [133].

Regarding starch film preparation with functional properties, it could be mentioned the rye starch films containing rosehip extract (1%). It showed high antioxidant properties and the extract increased film elasticity. For preventing lipid oxidation, this film was tested in chicken breast wrapping. The production of radical species in the chicken sample was reduced by 50% regrading to control treatment on the 9th day [134]. Similar results were obtained in a starch film containing clove leaf oil for the packaging of cheese [135]. The addition of plant oil resulted in good film radical-scavenging activity and antimicrobial activity against *Listeria monocytogenes* as well. In addition, the concentration of oxidant compounds in cheese was reduced by 54%, preventing lipid oxidation. In another work, tapioca starch films loaded with carvacrol were studied for potential use in food packaging [136]. Carvacrol decreased the film tensile properties and swelling degree but WVP and solubility increased. Good antioxidant and antimicrobial properties of the films were demonstrated suggesting this product could be used as a bioactive film. Starch packaging films, containing different bioactive compounds such as gallic acid, chitosan, and carvacrol were also prepared [137]. Starch films were used as antimicrobial packaging to control foodborne pathogens and meat microbiota in ham. Films containing chitosan and carvacrol showed higher activity than films with gallic acid. Cassava starch films carrying rosemary antioxidant extracts were also studied [138], provided high antioxidant and UV-barrier properties in the film. Total polyphenols were migrated in an aqueous food simulant, but a small amount was found in the fatty food after 1 week of exposure. The natural extract increased the starch film integrity in composting assays. The films containing cowpea starch and maqui berry extract were tested as a biodegradable active film to protect Salmon [139]. Films showed high antioxidant and UV-barrier properties. Salmon pieces were wrapped in those films and were protected against lipid oxidation. It has been reported that starch-based coatings have also been successfully used to extend the shelf life of fruit and vegetables such as banana [140]. Moreover, sweet potato starch was used as a coating of shrimps during cold storage successfully [141]. This starch combined with thyme essential oil (4%) was used for egg preservation [142]. It kept the quality of the eggs for 2 weeks more than non-coated samples.

During recent years, intelligent films as a sensor for food quality have been developed. The films based on starch/PVA incorporated with betalains-rich red pitaya peel extract were prepared [129]. The antimicrobial, antioxidant, and barrier properties were improved due to the addition of the extract. This film was designed to sense the NH_3_ content produced in shrimp during storage. Film color changed upon volatile *N*-compound accumulation, and pH change to alkaline values due to the shrimp spoilage, indicating it could be used as an indicator of food quality. Other pH-sensitive packaging films were developed based on cassava starch and L. ruthenicum anthocyanins for monitor the pork freshness [143]. Starch films exhibited remarkable color variations with the quality change of pork.

Finally, different starch aerogels systems were studied, including their production using scCO_2_ [144]. These aerogels could be impregnated with active materials such as quercetin [145]. The obtained material showed good dissolution time and the lowest burst-like effect. However, an effective decrease of the quercetin release occurred using the aerogels. It has been demonstrated that the aerogel structure and performance in terms of thermal conductivity ad mechanical properties could be improved by adding natural fibers [97,146]. Besides, aerogels were impregnated with the antifungal substance trans-2-hexenal [147]. Chemical crosslinking allowed the sustained release of the volatile compound, which in turn led to slower lethality of Aspergillus cells inoculated on pistachio nuts compared with the non-coated condition. Poorly water-soluble vitamins (vitamin E and K_12)_ were also impregnated in starch aerogels. Buffer release assays demonstrated an improvement in starch and aerogels release [148]. All this work demonstrates the potential and versatility of using starch polysaccharide in food packaging applications.

### 3.2. Microbial Exopolysaccharides

Microbial exopolysaccharides (EPSs) have several advantages as compared to aforementioned plant and marine polysaccharides. They are easier to produce on industrial level, since growth of microorganisms does not depend on regional and climatic conditions, or marine pollution [149]. Furthermore, microorganisms exhibit high growth rate and usually secrete EPS into the extracellular environment, facilitating the recovery of the final product [150,151]. However, they are more expensive than the plant-based ones, primarily due to production costs [149].

Generally, microbial EPSs are produced by fermentation process. Properties of polysaccharide such as molecular mass, solubility, chemical structure, etc. are strain dependent and strongly affected by culture conditions. Therefore, optimization of fermentation process and selection of high-producing strain are carefully considered for industrial production. The most important parameters to be optimized include composition of growth medium, temperature, pH, aeration [151,152]. Growth medium for EPS production can be chemically defined as well as low cost media based on various food and/or agricultural wastes or byproducts and it is designed to have high carbon-to-nitrogen ratio [149,152]. Many microorganisms require different conditions regarding temperature and pH for growth and EPS production. Therefore, it may be required that culture conditions may be changed after defined time needed for maximum biomass reaching to the conditions favorable for polysaccharides synthesis [152].

The isolation of EPS from fermented medium is usually done by precipitation after removal of cells. Obtained EPS is then subjected to further purification [153,154,155,156].

Microorganisms produce a wide variety of diverse EPSs and their applications in different industries are numerous. Microbial EPSs used for food packages preparation are only discussed here in more details.

#### 3.2.1. Pullulan

**Figure 10:**
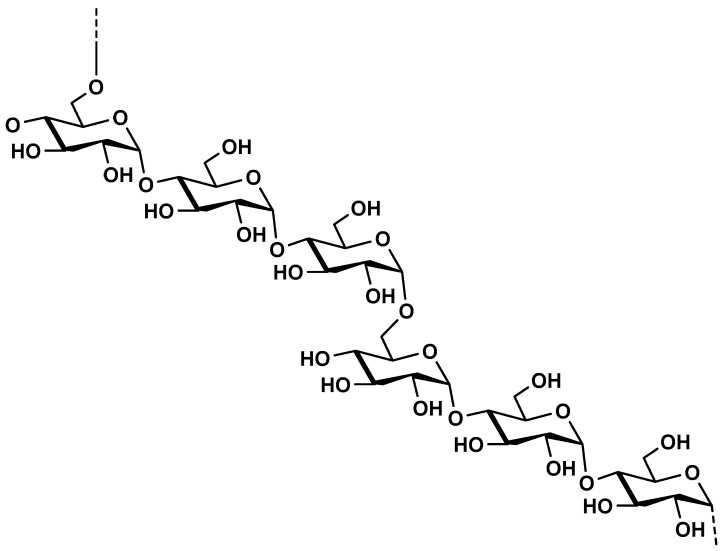
Chemical structure of pullulan.

Pullulan is a homopolysaccharide obtained from the fermentation medium of the fungus-like yeast *Aureobasidium pullulans*. It consists of maltotriose repeating units connected to each other by *α*-(1→6) glycosidic bonds. Glucose molecules within each maltotriose are linked by *α*-(1→4) linkages (Figure 10). This structure provides great flexibility of pullulan molecules, lack of crystallinity and high solubility in water [157]. The molecular weight of pullulan varies depending on the strain, culture conditions, and time of fermentation, and it is between 10 and 400 kDa [109].

Pullulan-based materials have great mechanical strength, high barrier toward oxygen and CO_2_, and high adhesive ability and capacity to form fibers and films. The viscosity of pullulan solution is independent on temperature, pH, and presence of most metal ions including sodium chloride [157,158]. Trinetta et al. showed that glycerol has significant influence on optical and mechanical properties of pullulan edible films [159]. To increase water vapor barrier, pullulan films are generally combined with proteins [160] and/or waxes [161,162]. Cozzolino and coworkers developed the pullulan/silica nanoparticles coatings and pullulan/microfibrilated cellulose/borax onto bi-orientated polypropylene films, as superficial materials with high barrier to oxygen and CO_2_, which allowed the use of these coatings in MAP food packaging [163,164]. There were several approaches to introduce antioxidant and antimicrobial activity of pullulan films. Fernandes et al. modified pullulan by grafting the with 3-aminopropyltrimethoxysilane, to obtain transparent food packaging films with high biological activity toward *S. aureus* and *E. coli* [165]. Addition of lysosome nanofibers into pullulan matrix proved high antibacterial activity against *S. aureus* [166]. Tea polyphenols incorporated into pullulan/carboxymethyl cellulose electrospun led to improved quality of coated strawberries during the storage [167]. Pullulan coatings enriched with extracts from *Bergenia crassifolia* showed high antibacterial activity toward various Gram-positive and Gram-negative foodborne pathogens and prolonged the shelf life of peppers and apples during postharvest storage [168]. Pullulan/*Laminaria Japonica* derived oligosaccharides coatings prolonged the shelf life of cherry tomatoes [169]. Morsy et al. [170] investigated the effect of pullulan coating that contained nisin on the quality and shelf life of chicken eggs during storage at room and refrigeration temperatures. The results showed that pullulan coatings with or without nisin minimized weight loss and maintained the albumen and yolk quality of eggs for 3 and 4 weeks longer than that of non-coated eggs during storage at 25 °C and 4 °C, respectively. However, nisin significantly improved antimicrobial properties of pullulan coatings. After 5 weeks of storage at room temperature, the number of total viable bacterial counts reached 8.03 log10 CFU/g for uncoated eggs while pullulan and pullulan-nisin coated eggs showed 5.61 log10 CFU/g and 5.05 log10 CFU/g, respectively: numbers of psychrophilic bacteria were 4.93 log10 CFU/g, 3.73 log10 CFU/g, and 3.07 log10 CFU/g and yeasts and molds counts were 6.03 log10 CFU/g, 5.08 log10 CFU/g, and 3.56 log10 CFU/g for uncoated, pullulan-coated, and pullulan-nisin coated eggs, respectively. Zhang, Wei and Han [171] developed antibacterial film by incorporating capsaicin into sodium alginate/pullulan composite films which extended the shelf life of fresh-cut apples for 3 days. Guo et al. [172] developed intelligent double-layer mats based on electrospun fibers of pullulan and zein intended for food freshness monitoring and preservation. To obtain colorimetric-responsive layer (with a pH-sensing range from 2–12) purple sweet potato extract was added to pullulan and antibacterial layer was composed of zein, glycerol, and carvacol, while both layers possessed antioxidative activity. The mats extended the shelf life of pork by 24 h at 25 °C.

#### 3.2.2. Kefiran

**Figure 11:**
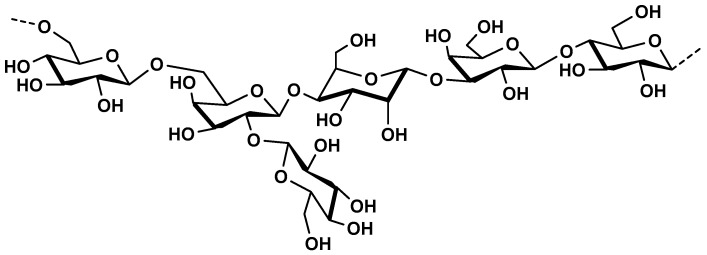
Chemical structure of Kefiran.

Kefiran is another EPS, produced by lactic acid bacteria. It is the main polysaccharide component of kefir grain, the starter culture for preparation of milk-based fermented beverage [173]. It is heteropolysaccharide made up of glucose and galactose in equimolar quantities (Figure 11).

Kefiran consists of repeating branched hexa- or heptasaccharide units which are composed of a regular pentasaccharide unit to which one or two sugar residues are randomly linked [174]. The molecular weight of kefiran is reported to be in the range of 10^5^ to 10^7^ Da, depending on the source, as well as purification method [156,175,176].

Kefiran is water-soluble polysaccharide which exhibits Newtonian behavior in diluted solution, while pseudoplastic behavior is observed at higher concentrations [176]. An advantage of kefiran compared to other polysaccharides is its antimicrobial activity [149]. It was shown that kefiran films plasticized with glycerol possessed better physical, mechanical and water vapor barrier properties than kefiran films plasticized with sorbitol [177,178]. On the other hand, oleic acid had plasticizing effect on kefiran and significantly improved water vapor barrier but diminished mechanical strength [179]. Other approaches to improve mechanical, water vapor barrier and antimicrobial properties were blending with carboxymethyl cellulose or starch and synergetic effect by incorporation of different metal oxide nanoparticles, such as ZnO and CuO [124,180,181]. Rad, Sharifan and Asadi [182] investigated physico–chemical and antimicrobial properties of kefiran/waterborne polyurethane blend film incorporated with different concentrations (5, 10, 15 and 20% (v/v)) of Zataria multiflora and Rosmarinus officinalis essential oils intended for ostrich meat packaging. The antibacterial activity of the films against *Escherichia coli*, *Staphylococcus aureus*, and *Lactobacillus plantarum* was obtained with all concentrations of both essential oils. By wrapping the ostrich meat with kefiran/waterborne polyurethane films incorporated with 20% Zataria multiflora and Rosmarinus officinalis essential oils significant reduction of total viable cells count was achieved during 12 days of storage at 4 °C. Sensory evaluations of ostrich meat samples revealed prolonged acceptance of coated samples for 6 days more, compared to uncoated control.

### 3.3. Marine Polysaccharides

As described above, polysaccharides are abundantly present in lot of biomasses such as plant and microorganisms. If it is considered marine biotopes, seaweeds and crustaceans are the most important sources of polysaccharides [183,184]. Consequently, the last 2 decades lots of marine polysaccharides such as chitosan, alginate, agar–agar, and carrageenan have been largely described for their techno-functional applications. In this paragraph it will be summarized the mains uses of these very interesting polysaccharides in food packaging topic.

#### 3.3.1. Chitosan

**Figure 12:**
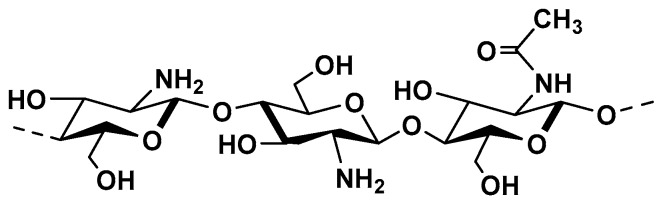
Chemical structure of chitosan.

Chitosan (CS) (Figure 12) is a polysaccharide made up with two different monomers *N*-acetylglucosamine and glucosamine linked through *β*-(1→4)-glycosidic bonds [185].

This natural polysaccharide is derived from another polysaccharide called chitin, which is one of most earth abundant polysaccharide after cellulose. As largely described in the literature, chitin is present in a huge range of living organisms such as crustaceans, but also in insects and fungi. Chitin and chitosan are generally characterized by the degree of acetamidation/acetylation (DA) and expressed as a percentage of acetamide groups present. Therefore, for chitin this DA is greater than 50% and less than 50% for chitosan [186,187,188]. Chitin is an insoluble polysaccharide in water or other common solvents. Chitin can be converted into CS under high alkaline and hot conditions. After this hard-chemical treatment, produced chitosan become soluble in acidic medium (HCl, acetic acid, lactic acid…). Chitosan and its derivatives have numerous applications such as biomaterial for production of tissues, skin and other materials [183,187,188,189].

Several recent studies have shown that it is possible to use chitosan for food packaging, due to its physico–chemical properties [184]. In a very interesting review, Fernandez-Saiz [190] gave an overview of the food packaging applications using chitosan. As mentioned by author, the main advantages of chitosan are: (i) biocompatibility, (ii) non-toxicity, (iii) edibility, (iv) bioadhesivity and (v) biodegradability. CS films have a selective permeability to gases such as O_2_ and CO_2_ and good mechanical properties. Nevertheless, lots of studies well-shown that CS film has high water sensitivity which reduce barrier properties [183,190]. Consequently, several strategies were proposed, in terms of blending CS with: (i) polymers such as cellulose, polyethylene terephthalate (PET), gelatin, polyvinyl alcohol (PVA) and (ii) citric acid, glycerol, genipin as plasticizers and cross-linkers in order to enhance water-resistance while maintaining high biodegradability [191,192,193,194]. Moreover, it was particularly described the excellent coating and film-forming abilities coupled with a very good intrinsic antimicrobial property against fungi and bacteria as largely described in the literature [183,184,195,196]. In recent years, all these features were exploited by lots of research groups using CS in packaging field and more especially in the development of innovative and very promising active food packaging such as antimicrobial packaging [197,198]. For example, chitosan was reversible coated onto PET meat packaging to increase antimicrobial properties against meat pathogens such as *Escherichia coli*, *Salmonella enterica*, *Listeria monocytogenes* and *Candida albicans* [191]. In 2007, Duan et al. [199] used CS-lysozyme film to efficiently enhance microbial safety of mozzarella cheese against *Escherichia coli*, *Pseudomonas fluorescens*, or *Listeria monocytogenes*. It was shown that coated cheese had 0.43- to 1.25-, 0.40- to 1.40-, and 0.32- to 1.35- log reductions in *E. coli*, *P. fluorescens*, and *L. monocytogenes* in inoculated cheeses, respectively. Generally, combinations of chitosan with various essential oils have been investigated as coatings for meat, and fish-products. For example, chitosan edible films coated with essential oil from *Thymus piperella*, *Thymus moroderi*, *Citrus bergamia* enhanced food safety (antimicrobial and antioxidant properties) and the shelf life of cooked cured ham [200] and inhibited the growth of *Penicillium italicum* [201]. More, it was shown that regarding mechanical and optical properties, the incorporation of *Citrus bergamia* essential oil in chitosan films decreased the water vapor permeability and allowed the synthesis of composite edible films with less glossy, less deformability, and less resistance to break. Vasilatos et al. combined chitosan with rosemary oil and demonstrated that this coating prolonged the shelf life of turkey meat for 11 days [202]. Inclusion of rosemary oil in chitosan/montmorillonite matrix showed to retard chicken poultry lipid oxidization, discoloration and microbial spoilage [203]. On the other hand, chitosan incorporated with thyme oil [204], cinnamon oil [205] and clove oil [206] proved to prolong the shelf life of various sorts of fishes.

Generally, in the food industry neat chitosan has appeared increasingly studied due to its antimicrobial properties, especially as a coating on fruit. Obianom et al. demonstrated that 1.5 wt.% solution of chitosan could reduce the incidence of stem-end rot and anthracnose in inoculated and naturally infected avocados, hence significantly influenced the postharvest decay control of avocado [207]. Silva at al. proved that chitosan solutions could significantly delay ripening of guava fruit, by suppression of the respiratory rate, fresh weight loss, firmness, and skin color, with delay in the degradation of chlorophyll [208]. Pasquariello et al. showed that 0.5 wt.% solution of chitosan inhibited polyphenol oxidase and guaiacol peroxidase involved in sweet cherry fruit-browning processes, and enhanced antioxidant enzyme activities that reduce oxidative damage [209]. Drevinskas et al. investigated the influence of chitosan molecular weight on quality parameters of three different cultivars of kiwifruit. They demonstrated that coatings made from high molecular weight chitosan were efficient in prolonging the shelf life of Sentiabrskaya and Anykšta kiwifruit, whereas coatings made from low molecular weight chitosan were efficient for VIR kiwifruit [210].

Finally, it is important to mention the recent work from Wu et al. [211] where it was proposed a novel intelligent film based on immobilization of black rice bran anthocyanins onto chitosan matrix. This study revealed that these intelligent chitosan films grafted with anthocyanins exhibited very excellent: pH sensitivity character, antioxidant properties, and UV-barrier effect. As a main application, authors proposed to develop these chitosan edible films as intelligent food packaging to control the spoiling of shrimp and fish by monitoring the color changes of edible films. In addition, Takeshita and Yoda prepared translucent chitosan aerogels by the crosslinking gelation with formaldehyde and subsequent CO_2_ super critical drying [212]. The obtained aerogels consisted of entangled nanofibers with a high porosity up to ~97%, with visible transparency and low thermal conductivity of ~0.022 W m^−1^ K^−1^. The chitosan aerogels also possessed higher mechanical toughness than conventional silica aerogels. The characteristic mechanical properties associated with a low thermal conductivity presented a new category of thermal insulators—environmentally friendly, and transparent—that could potentially serve as layer in food packaging of temperature-sensitive food.

#### 3.3.2. Alginate

**Figure 13:**
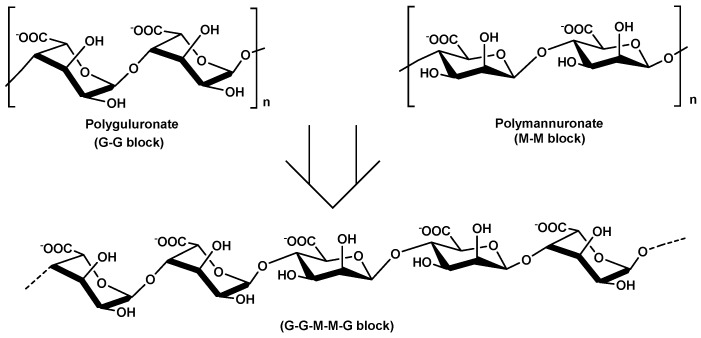
Mains chemical structure of seaweed alginates.

Alginate is mainly extracted (20–40%content of the dry weight) from the cell walls of brown marine seaweed (Phaeophyceae) such as *Ascophyllum nodosum*, *Laminaria* species, *Fucus* species, *Macrocystis pyrifera* and *Ecklonia* species [213,214,215] and in low amount extracted from bacterial origin as exopolysaccharides such in the case of *Azetobacter* and *Pseudomonas* species [213]. Chemically, alginates are made up of (1→4)-linked *α*-l-guluronic acid (G) and *β*-d-mannuronic acid (M). According to the brown algae species, guluronic and mannuronic acids are arranged in homogeneous blocks (GG or MM) and heterogeneous blocks (GM or MG) blocks which allow the presence of very different chemical alginate structures with several molecular weights and variable physico–chemical properties (Figure 13).

Mostly, the main important structural characteristics of alginates are: M/G ratio, molecular weights, diad frequencies (F_GM_, F_MG_, F_GG_ and F_MM_) and monad values (F_G_ and F_M_). All this structural information are strongly connected with rheological properties on alginate solutions [216,217]. As generally well-described, GG blocks provide more rigid conformation of alginate structures, whereas MM blocks give flexibility and linearity of alginate backbone structure. Alginates with higher GG blocks give higher viscous solutions. Lots of industrial applications of alginates are based on hydrogel formation (strong gels), due to their capacity to bind divalent cations such as Ca^2+^ or Mg^2+^ with crosslinking processes. Of course, the gelling properties depend on alginate structure, the type of calcium/magnesium salt and their diffusions and the degree of sodium alginate conversion to calcium/magnesium alginate.

In the last decade, lots of studies investigated the use of alginate for food packaging development. Alginate edible films are essentially transparent, uniform and highly water-soluble [213]. To increase the water insolubility, ionic interactions are induced by using divalent cations such as Ca^2+^ to cross-link alginate during film synthesis due to ionic interactions and hydrogen bonding [218,219]. Therefore, for food packaging applications, coatings, and films could be typically produced from fast calcium reaction with sodium alginate solution, allowing intermolecular associations that generally involve the GG blocks. As largely mentioned in the literature, alginates have very good filmogenic properties, but need to be plasticized with glycerol or sorbitol, for example, to reduce brittleness after the drying process [220]. Jost et al. [220] clearly showed that alginate films properties are related to plasticizer (concentration and type). Glycerol and sorbitol improved the flexibility of alginate films, but only glycerol seems to increase the water vapor and oxygen permeability of alginate films. In some works, it was demonstrated that alginate coating allowed reduction of lipid oxidation, increase of moisture and enhance flavor, quality and texture of food [219]. Interestingly, coating of alginate/citric acid using a spraying process increased shelf-life extension by 5 to 7 days of sliced carrot [221]. In other study, enhancing of shelf life of bream fish (*Megalobrama amblycephala*) was observed using alginate-calcium coating containing tea polyphenol or ascorbic acid [222]. In general, addition of nano-reinforcement agents as whiskers cellulose can improve the mechanical (tensile strength, elastic modulus…) properties and water vapor barrier of alginate films, as in the case for example of alginate-acerola puree-based edible films [223]. Principally, authors mentioned that these films could be applied as edible coatings for numerous foods such as vegetables and fresh fruit to simply extend shelf life. It was pointed out by lots of studies that addition of bioactive agents such as antioxidant or antimicrobial to alginate edible films is more beneficial by comparison with the industrial process using direct application of bioactive agent in food [224,225]. Therefore, a large diversity of antimicrobial agent including chemical and natural (essential oil, phenolic compounds…) have been efficiently incorporated in alginate film for food packaging development [226,227,228,229]. For example, it can be mentioned recent works incorporating antioxidants and antimicrobial agents for food applications in alginate films and coating such as: trans-cinnamaldehyde [230]; carvacrol, methyl cinnamate [231]; pomegranate peel extract [232]; thyme oil [233]; acetic acid/lactic acid [234,235]; nisin/EDTA [236]; resveratrol [237]; tea polyphenols [238]; garlic oil [239]; ascorbic acid [240]; lycopene [241]; clove essential oil [242] and tocopherols [243]. In a similar approach, some other food ingredients have been incorporated such as antibrowning molecules. In fact, food color is proving of quality and browning reactions occur during shelf life of vegetables, meats, and fruit. These colorations generally result from enzymatic or non-enzymatic oxidation of phenolic compounds (vegetables and fruit) or proteins (meat) [244,245,246,247]. In this context, as described in the literature, it can be suggested the use of: (i) citric acid, ascorbic acid, lemongrass essential oil and thyme essential oil into alginate coating of fruit such as apples and pineapples [248,249] and (ii) glutathione or *N*-acetylcysteine into alginate coating of apples and pears [248,250,251,252]. Nevertheless, it is important to mention that some studies revealed that the more important limiting factor for using antioxidant/antimicrobial/antibrowning agents onto alginate film/coating food packaging could be the appearance of strong flavor for food ingredient [213,226].

Franco et al. proposed new antimicrobial aerogel food packaging, by adsorption of antioxidant compound quercetin on alginate aerogel by CO_2_ supercritical technology [145]. Migration tests using food simulant showed that the dissolution rate of quercetin loaded on aerogels was significantly lower than pure quercetin. Thus, the supercritical technology proved to be suitable to obtain controlled-release systems that potentially could be used as active layer of food package with enhanced properties. Moreover, there are numerous proofs in the literature that alginate is suitable matrix for encapsulation of bioactive substances such as vitamins [253], proanthocyanidins [254], phytochemicals [255]. In addition, CO_2_ supercritical drying provide their controlled release from aerogels in desired time. Although alginate-based aerogels containing bioactive compounds were mostly studied for wound healing and drug delivery applications, ability to release active component in specific simulants confirmed that these materials have potentials to be used in the food packaging industry, as an active interior layer of package/container for transports of food products, where thermal insulation of material and release of active antimicrobial within prolonged time is essential to maintain the quality of food products.

#### 3.3.3. Agar and Carrageenans

**Figure 14:**
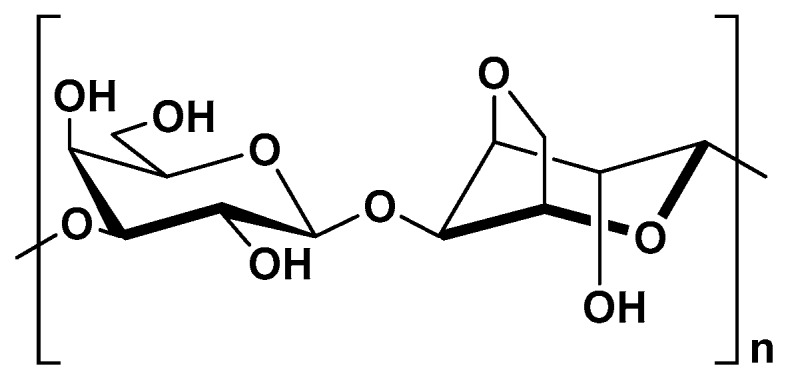
Chemical structure of agar.

From red marine macroalgae, it could be talked about galactan as a family of polysaccharides mostly signified by carrageenans and agars, which are largely explored in food and pharmaceutical industries due to interesting rheological and biological properties [256,257,258,259]. Agar is principally extracted from the cell walls of red algae from Rhodophyceae class such as *Pterocladia*, *Gracilaria* or *Gelidium* species [256,259]. Generally, agars are derived from agarose and a mixture of heterogeneous charged galactans called agaropectin [257]. Agarose, the gelling part of agar is composed of 3,6-anhydro-*α*-l-galactose and *β*-d-galactose repeating units (Figure 14).

The excellent gelling and film properties of agar allow broadly uses in pharmaceutical and food applications. In fact, in recent decades lots of applications of seaweed-based polysaccharides in food packaging and coatings were investigated using agar. Agar is extensively used thanks to its availability, gelling properties, encapsulation efficiency and biocompatibility. Basumatary et al. [260] studied agar-based nanocomposites incorporated with silver nanoparticles (AgNPs) as antimicrobial agent for food packaging development. In this interesting work, authors used diverse *Lagerstroemia speciosa* aqueous extracts to the synthesis AgNPS in good yield. These agar composite films had very good antimicrobial effect against *Aeromonas hydrophilla*, a Gram-negative foodborne pathogen. Films possessed improved mechanical (elongation at break) and appearance properties but tensile strength decreased by addition of AgNPs into agar-based film. In a same strategy, Shukla et al. [261] proposed agar-based silver nanocomposites films with antimicrobial applications for food. In other approaches, agar-nano-cellulose-savory essential oil-based composite films were proposed as active packaging in order to improve the shelf life and safety of foodstuff [262]; and nanoclays were used to develop biodegradable food packaging with enhanced water vapor barrier and mechanical properties [263]. More, it was reported that unpurified agar-based extracts from *Gelidium sesquipedale* seaweed could be easily use as bio-based food packaging film [264]. Due to impurities such as proteins and other polysaccharides (Floridean starch), these edible films were flexible without addition of plasticizer (glycerol…) and possess good water barrier (resistance to humidity conditions) and mechanical performance. Recently, functional agar films incorporated with curcumin were produced by casting method. These composites have efficient: (i) antioxidant and antibacterial activities, (ii) swelling ratio, (iii) water vapor barrier and (iv) UV-blocking properties [265]. Same authors used melanin nanoparticles from the sepia ink to synthetize antioxidant agar-based films with enhanced hydrophobicity, UV-blocking, water vapor barrier and mechanical properties [266]. Moreover, in situ mineralization of Cu- and Zn-nanoparticles into agar matrix showed high UV-blocking effect, with excellent antimicrobial activity toward different bacteria and fungi [19,267,268]. Generally, it was shown, as similar to alginate composites, that agar-starch-based edible films possessed very good mechanical properties for food packaging development [269,270]. In addition, agar/starch films in combination with anthocyanin extracts from purple sweet potato can serve as an intelligent pH sensor to monitor spoilage of pork [25].

Finally, it could be noticed some recent works using: (i) agar and agar-gelatin-based edible film incorporating green tea extract for antioxidant and antimicrobial activities [271], (ii) agar-ZnO nanocomposite films to increase the shelf life of green grape [272], (iii) agar-*stevia rebaudiana* extract edible films [273] and (iv) agar-silver nanocomposites for food packaging able to increase the shelf life of fresh chicken stored at 4 °C [274].

**Figure 15:**
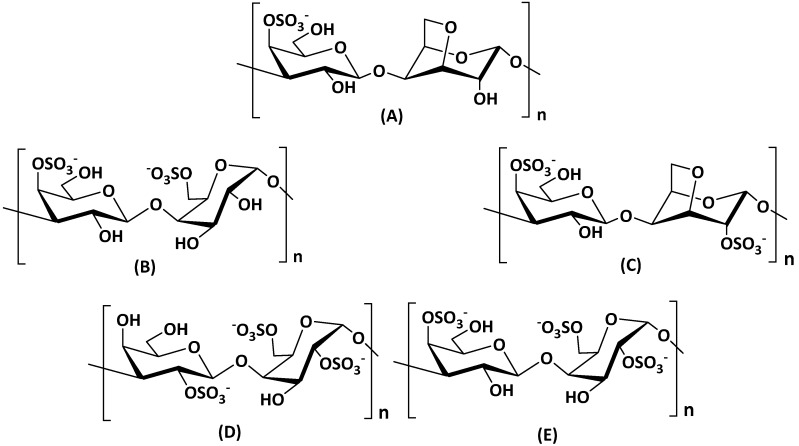
Main chemical structures of carrageenans. (**A**) κ-carrageenan, (**B**) μ-carrageenan, (**C**) ι-carrageenan, (**D**) λ-carrageenan and (**E**) ν-carrageenan.

Carrageenans are sulfated galactans generally extracted from diverse red seaweeds from *Rhodophyceae family* such as *Chondrus* sp., *Eucheuma* sp. or *Gigartina* sp. [256,257,258,259] 19. As clearly defined by Delattre et al. [256], carrageenans are structurally similar to agar, excluding the presence of 3,6-anhydro-d-galactose instead of 3,6-anhydro-l-galactose in agar. As well-defined (Figure 14), carrageenans are constituted of linear galactans made up of d-galactose residues α-(1,4) and β-(1,3)-linked alternately and are composed of different families and subfamilies such as: κ-carrageenan, λ-carrageenan, μ-carrageenan, ι-carrageenan, and ν-carrageenan (Figure 15).

Carrageenans are generally used as stabilizing, gelling, and emulsifying ingredients in food, dairy, and pharmaceutical industries [256,275]. More, as natural highly hydrophilic polysaccharides, these sulfated galactans offer high film-forming capabilities due to excellent gelling ability of carrageenans [276,277]. In fact, as reported by Karbowiak et al. (2006) when it was cooled a hot carrageenan solution during the film casting process, it was observed during the drying step a conformational change of random coil of polysaccharides to a double helix formation, which led to the production of a very compact film with good structural and mechanical properties [276]. Consequently, carrageenan found lots of applications in the food industry. As for example, Varela and Fiszman (2011) described that carrageenan films presented good oxygen barrier allowed a very good protection against lipid oxidation [278]. Moreover, antimicrobial and antioxidant carrageenan films were proposed for food application as active packaging using composite with: (i) potassium sorbate [279], (ii) essential oil with high number of phenolic compounds from *Satureja hortensis* a medicinal plant composed mostly found in Mediterranean [280], (iii) *Zataria multiflora Boiss* and *Mentha pulegium* essential oils [281] or (iv) pomegranate seed oil [282]. In these studies, it was particularly shown by authors that the addition of essential oils into carrageenan films significantly improved the water vapor barrier properties and the films were opaque and more flexible. In other work, plant extract from *Zataria multiflora* was incorporated in κ-carrageenan/nanoclay composite films to improve UV protection, mechanical properties, and antimicrobial effect against *B. cereus*, *E. coli*, *S. aureus* and *P. aeruginosa* [282]. Finally, some works revealed that multilayer films based on κ-carrageenan/agar/clay nanocomposite mixed with polylactide (PLA) bioplastic or cellulose nanocrystal (CNC) improved barrier, optical, thermal stability and mechanical properties for food packaging field [283,284].

## 4. Conclusions

Food products follow the progress in the existing one’s packaging processes, and the application of new achievements in packaging area. The choice of packing material is very complex since consideration should be given to the type of food product, its sustainability, and environmental conditions. Since commonly used synthetic packaging materials are facing environmental issues, an increasing production of bio-packages is expected in the future, which could completely replace the synthetic packaging on the market. Moreover, polysaccharide-based materials promise to expand the future of edible films, and intelligent and active sensors in food packaging, due to proven gas barrier properties and biological activity. The main disadvantage of polysaccharide-based materials is their sensitivity to moisture and mechanical strength, requiring addition of one more component in the system, which can increase the cost of final product. However, with the increasing awareness of need for sustainable green technologies and products on the market, the prices of raw biomaterials, and among them of polysaccharides, starts decreasing. It is foreseen the growth of development and application of polysaccharide multifunctional materials in the food packaging sector in the future.
